# The Proliferation of Dentate Gyrus Progenitors in the Ferret Hippocampus by Neonatal Exposure to Valproic Acid

**DOI:** 10.3389/fnins.2021.736313

**Published:** 2021-09-28

**Authors:** Kazuhiko Sawada, Shiori Kamiya, Ichio Aoki

**Affiliations:** ^1^Department of Nutrition, Faculty of Medical and Health Sciences, Tsukuba International University, Tsuchiura, Japan; ^2^Department of Molecular Imaging and Theranostics, National Institutes for Quantum Science and Technology, Chiba, Japan; ^3^Institute for Quantum Life Science, National Institutes for Quantum Science and Technology, Chiba, Japan

**Keywords:** hippocampus, development, valproic acid, ferret, immunohistochemistry

## Abstract

Prenatal and neonatal exposure to valproic acid (VPA) is associated with human autism spectrum disorder (ASD) and can alter the development of several brain regions, such as the cerebral cortex, cerebellum, and amygdala. Neonatal VPA exposure induces ASD-like behavioral abnormalities in a gyrencephalic mammal, ferret, but it has not been evaluated in brain regions other than the cerebral cortex in this animal. This study aimed to facilitate a comprehensive understanding of brain abnormalities induced by developmental VPA exposure in ferrets. We examined gross structural changes in the hippocampus and tracked proliferative cells by 5-bromo-2-deoxyuridine (BrdU) labeling following VPA administration to ferret infants on postnatal days (PDs) 6 and 7 at 200 μg/g of body weight. *Ex vivo* short repetition time/time to echo magnetic resonance imaging (MRI) with high spatial resolution at 7-T was obtained from the fixed brain of PD 20 ferrets. The hippocampal volume estimated using MRI-based volumetry was not significantly different between the two groups of ferrets, and optical comparisons on coronal magnetic resonance images revealed no differences in gross structures of the hippocampus between VPA-treated and control ferrets. BrdU-labeled cells were observed throughout the hippocampus of both two groups at PD 20. BrdU-labeled cells were immunopositive for Sox2 (>70%) and almost immunonegative for NeuN, S100 protein, and glial fibrillary acidic protein. BrdU-labeled Sox2-positive progenitors were abundant, particularly in the subgranular layer of the dentate gyrus (DG), and were denser in VPA-treated ferrets. When BrdU-labeled Sox2-positive progenitors were examined at 2 h after the second VPA administration on PD 7, their density in the granular/subgranular layer and hilus of the DG was significantly greater in VPA-treated ferrets compared to controls. The findings suggest that VPA exposure to ferret infants facilitates the proliferation of DG progenitors, supplying excessive progenitors for hippocampal adult neurogenesis to the subgranular layer.

## Introduction

Valproic acid (VPA), a well-known antiepileptic/anticonvulsant drug, also acts as an inhibitor of histone deacetylases 1 and 2 (Göttlicher et al., 2001; Phiel et al., 2001). Many studies have reported autism spectrum disorder (ASD)-like behavioral abnormalities induced by VPA exposure during prenatal and neonatal periods in various mammalian species such as mice (Yochum et al., 2008; Burenkova et al., 2014), rats (Miyazaki et al., 2005; Mychasiuk et al., 2012; Favre et al., 2013), common marmosets (Yasue et al., 2018), and ferrets (Krahe et al., 2016). These behavioral abnormalities are believed to be associated with cerebral cortical abnormalities caused by developmental VPA exposure (Sabers et al., 2014; Wood et al., 2014; Fujimura et al., 2016; Lauber et al., 2016; Sawada et al., 2021). In contrast, prenatal and neonatal VPA exposure can alter the development of other brain regions, such as the hippocampus (Edalatmanesh et al., 2013; Juliandi et al., 2015; Wang et al., 2016; Hou et al., 2018; Win-Shwe et al., 2018), cerebellum (Hou et al., 2018; Mirza and Sharma, 2019), and amygdala (Olexová et al., 2016; Wang et al., 2016). In association with such VAP-induced brain abnormalities, VPA regulates neurogenesis; however, studies have reported inconsistent findings – facilitation of neuronal differentiation of rat adult hippocampal neuronal progenitors (Hsieh et al., 2004) and mouse embryonic stem cells (Juliandi et al., 2012) but inhibition of neuronal differentiation of cortical progenitors in mouse fetuses (Fujimura et al., 2016).

Ferrets are small laboratory animals that have morphological characteristics of the brain similar to humans, not found in rodents: for example, the gyrencephalic cerebral cortex (Sawada and Watanabe, 2012; Kroenke et al., 2014) and torque asymmetry of the cerebellum (Sawada et al., 2015, 2020). VPA exposure to ferret infants decreased gyrification with thickened cerebral cortex at the sulcal floors (Sawada et al., 2021) that is comparable to the cortical abnormality observed in a type of human ADS (Ecker et al., 2013; Libero et al., 2014, 2019). Such gyrification abnormalities were involved in the excess production of cortical neurons by promoting neurogenesis or proliferation of cortical progenitors in ferrets (Sawada et al., 2021). The effect of neonatal VPA exposure, however, has not yet been evaluated in brain regions other than the cerebral cortex in gyrencephalic mammals such as ferrets. This study aimed to facilitate a comprehensive understanding of the brain abnormalities induced by developmental VPA exposure. The hippocampus is known as a target region by developmental VPA exposure in rodents, both morphologically and functionally (Edalatmanesh et al., 2013; Juliandi et al., 2015; Wang et al., 2016; Hou et al., 2018; Win-Shwe et al., 2018). Therefore, we examined gross structural and histological changes in the hippocampus and tracked proliferative cells by BrdU labeling following VPA administration to ferret infants on postnatal days (PDs) 6 and 7.

## Materials and Methods

### Animals

Fourteen male ferret pups naturally delivered by six pregnant ferrets were purchased from Japan SLC (Hamamatsu, Japan). The pups were reared with lactating mothers (4–6 pups/mother) in stainless-steel cages (80 cm × 50 cm × 35 cm) maintained at 21.5 ± 2.5°C under 12-h artificial illumination in the Facility of Animal Breeding, Nakaizu Laboratory, Japan SLC. All lactating mothers were fed a pellet diet (High-Density Ferret Diet 5L14; PMI Feeds, Inc., St. Louis, MO, United States) and tap water *ad libitum*.

Seven ferrets were intraperitoneally injected VPA on PDs 6 and 7 at 200 μg/0.01 ml/g body weight. 5-Bromo-2-deoxyuridine (BrdU; Sigma-Aldrich, St. Louis, MO, United States) was injected at 30 μg/0.01 ml/g body weight simultaneously with the last injection of VPA. As controls, seven ferrets were administered BrdU on PD 7. Two hours after BrdU injection, three pups in each group were perfused with 4% paraformaldehyde (PFA) in phosphate-buffered saline (PBS) under deep anesthesia with ∼2% isoflurane gas. The remaining four ferrets in each group were reared until PD 20 and then perfused with PFA under deep anesthesia.

### Magnetic Resonance Imaging Measurements and Magnetic Resonance Imaging-Based Analysis

Three-dimensional (3D) magnetic resonance (MR) images were acquired from the fixed brains of PD 20 ferrets using a 7.0-T magnetic resonance imaging (MRI) system (magnet: 400 mm inner diameter bore; Kobelco and Jastec, Kobe, Japan; console: AVANCE-I; Bruker BioSpin, Ettlingen, Germany) according to the procedure reported previously (Sawada and Aoki, 2017).

The hippocampus was segmented semiautomatically on coronal (transaxial) MRIs based on image contrast as well as user knowledge of anatomy using the “Morpho” tool of the SliceOmatic software ver. 4.3 (TomoVision, Montreal, QC, Canada), according to the procedure reported previously (Sawada et al., 2013). The segmented images were used to calculate the hippocampal volume and to render the hippocampus in 3D using SliceOmatic software, as reported previously (Sawada et al., 2013).

### Immunohistochemical Procedures

Cerebral hemispheres were immersed in 30% sucrose-PBS solution overnight and then embedded in optimal cutting temperature compound (Sakura Finetek Japan Co., Ltd., Tokyo, Japan) at −70°C. Coronal cryosections of the hemispheres at 100 μm thickness were made from PD 7 and PD 20 ferrets with or without neonatal VPA exposure using a Retratome (REM-700; Yamato Kohki Industrial Co., Ltd., Saitama, Japan) equipped with a refrigeration unit (Electro Freeze MC-802A, Yamato Kohki Industrial).

Immunofluorescence staining was performed on floating sections with identical staining conditions using the same sets of the solution by the slight modified procedures in the previous report (Sawada, 2019). The primary antibodies used were highly specific for ferret tissues and the endogenous antigen (BrdU) (Sawada, 2019; Kamiya and Sawada, 2021) and included rabbit antibodies to calbindin D-28k (1:1,000; CB38; Swant, Bellinzona, Switzerland), NeuN (1:1,000; ABN78; Millipore, Billerica, CA, United States), and S100 protein (1:200; 942001; Immunostar, Hudson, WI, United States); mouse antibodies to glial fibrillary acidic protein (GFAP) (1:1,000, G3893, Sigma-Aldrich); a goat antibody to Sox2 (1:1,000; AF2018, R&D Systems Minneapolis, MN, United States); a rat antibody to phospho-histone H3 (PH3) (1:1,000; ab10543; Abcam, Cambridge, United Kingdom); and a sheep antibody to BrdU (1:500; ab1893, Abcam). Secondary antibodies included Alexa 488 donkey anti-rabbit IgG (1:500; A21206, Thermo Fisher Scientific, Waltham, MA, United States), Alexa 555 donkey anti-mouse IgG (1:500; A31570, Thermo Fisher Scientific), Alexa 555 donkey anti-goat IgG (1:500; A-21432, Thermo Fisher Scientific), Alexa 647 donkey anti-sheep IgG (1:500; ab150179, Abcam), and Alexa 555 goat anti-rat IgG (1:500; A21434, Thermo Fisher Scientific). All sections were stained using Hoechst, mounted on slides, and coverslipped with glycerin.

### Estimation of Cell Density

Serial digital sectioning images (10 sections at 1 μm plane thickness) were obtained under a 20 × objective using an Axio Imager M2 ApoTome.2 microscope equipped with an AxioCam MRm camera (Zeiss, Gottingen, Germany) with Zen 2.3 blue edition software (Zeiss). Densities of immuno- and BrdU-labeled cells were calculated by the disector method using systematic random sampling, according to a previous report (Sawada, 2019). To estimate cell density, three consecutive sections at the coronal plane, including the caudal end of the splenium of the corpus callosum, were analyzed per animal. Frames with six square boxes (box size = 40 μm × 40 μm) were used to select systematically the region of interest (ROI) superimposed randomly on subregions of dorsal and/or ventral hippocampi. The percentage of immuno- or BrdU-labeled cells was estimated by summing the cells counted within all ROIs from six cerebral hemispheres of VPA-treated and control groups on PD 7, and eight hemispheres of both two groups on PD 20.

### Statistical Analysis

Data from the left and right hippocampi were considered to be independent samples, since no significant the left- and right-side differences in any measurements were demonstrated by a paired sample *t*-test. One-way analysis of variance (ANOVA) followed by Student’s *t*-tests was used to assess statistically the volume of the hippocampus between VPA-treated and control groups. A repeated-measures two-way ANOVA was used to assess the hippocampal region (dorsal or ventral)-related differences in immuno- and BrdU-labeled cell density. Scheffe’s test was performed as *post hoc* testing when significant interactions were revealed by two-way repeated-measures ANOVA, followed by simple main effects at *P* < 0.05.

The Chi-square test was conducted to compare the proportions of immunolabeled cells positive for each marker to BrdU-labeled cells between two groups of ferrets. The total number of BrdU-labeled cells counted was defined as “*n*” for the Chi-square test.

## Results

### Magnetic Resonance Imaging-Based Analysis of Postnatal Day 20 Ferret Hippocampi

**FIGURE 1:**
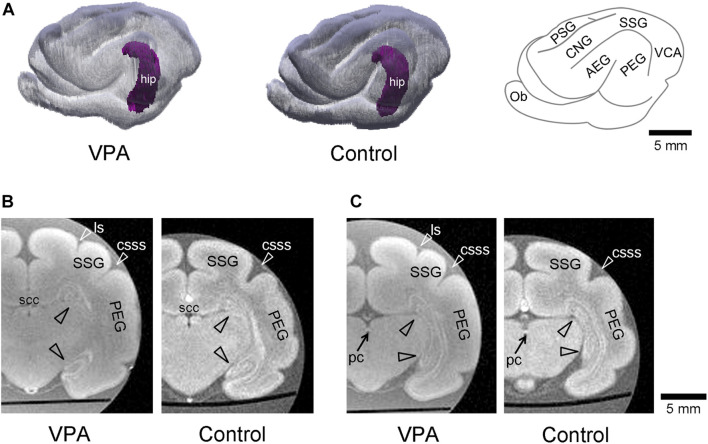
Three-dimensional volume-rendered images in cerebrum with the hippocampus of the postnatal day (PD) 20 ferrets. **(A)** The left and moderate images are reconstructed images of the cerebral cortex and hippocampus (hip; purple) of the left hemisphere in valproic acid (VPA)-treated and control ferrets, respectively. A gyral map of the left hemisphere is shown on the right. **(B)** Coronal *ex vivo* magnetic resonance (MR) images [using RARE sequence with short repetition time (TR) and minimum time to echo (TE) setting] of cerebrum including the hippocampus at the caudal end of the splenium of corpus callosum from VPA-treated ferret (left) and control one (right). **(C)** Coronal *ex vivo* MR images of cerebrum including the hippocampus at the posterior commissure from VPA-treated ferret (left) and control one (right). AEG, anterior ectosylvian gyrus; CNG, coronal gyrus; csss, caudal suprasylvian sulcus; Ob, olfactory bulb; ls, lateral sulcus; pc, posterior commissure; PEG, posterior ectosylvian gyrus; PSG, posterior sigmoid gyrus; scc, splenium of the corpus callosum; SSG, suprasylvian gyrus; VCA, visual cortical area.

The volume of the hippocampus was estimated based on MR images. The hippocampal volume was 27.3 ± 0.6 mm^3^ in VPA-treated ferrets, not significantly different from that in control ferrets (27.4 ± 0.6 mm^3^) (Table 1). Three-dimensional rendered images of the hippocampus with the cerebral cortex in the left hemisphere are depicted in Figure 1A. The hippocampus was situated in the deep portion of the posterior ectosylvian gyrus, corresponding to the auditory cortex. Gross structures of the hippocampus were not different between the VPA-treated and control ferrets. Coronal MR images of the hippocampus of VPA-treated ferrets and controls are shown in two identical planes, that is, the plane including the caudal end of the splenium of the corpus callosum (Figure 1B) and in the plane including the posterior commissure (Figure 1C). In coronal MR images at the caudal end of the splenium of the corpus callosum, the dorsal hippocampus began to descend in both groups (Figure 1B). A connection from the dorsal to ventral hippocampus was found in coronal MR images at the posterior commissure in VPA-treated and control ferrets (Figure 1C).

**TABLE 1:** Hippocampal volume in VPA-treated ferrets on PD 20.

	VPA (*n* = 8)	Control (*n* = 8)
Hippocampal volume (mm^3^)	27.3 ± 0.6	27.4 ± 0.6

*Data are represented as mean ± SEM of eight cerebral hemispheres.*

### Immunohistochemical Analysis of Postnatal Day 20 Ferret Hippocampi

**FIGURE 2:**
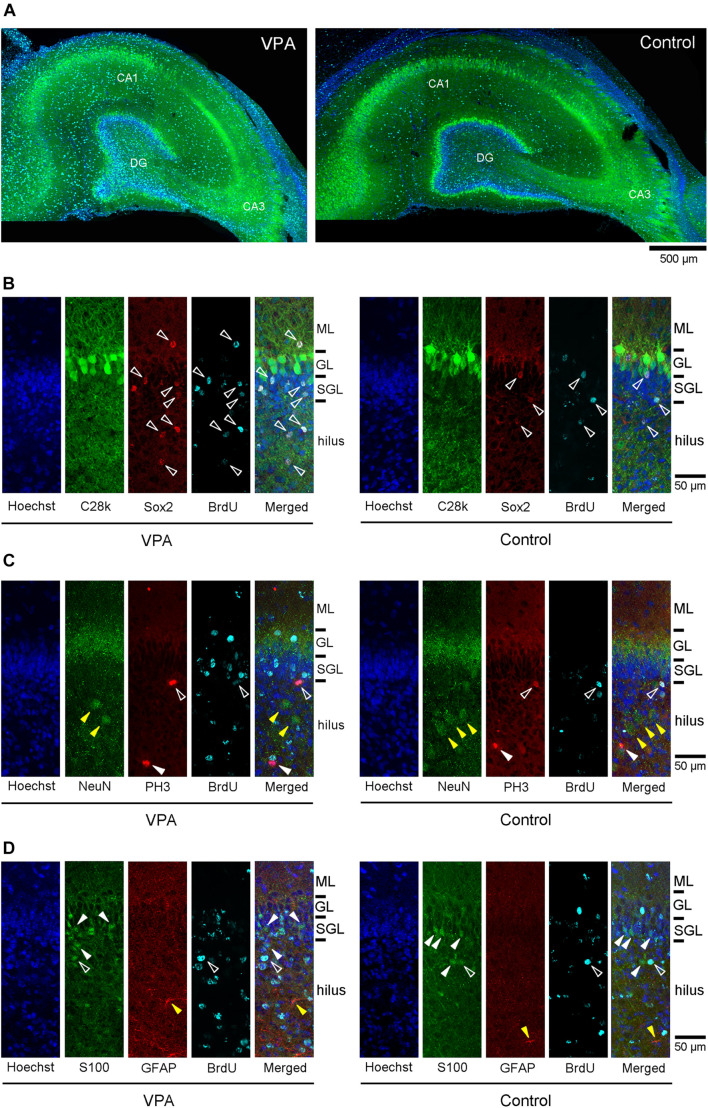
Immunofluorescence images for antibodies against various marker antigens with 5-bromo-2-deoxyuridine (BrdU)-labeling and Hoechst staining in dorsal hippocampi of postnatal day (PD) 20 ferrets. **(A)** Low-magnification images of calbindin D-28k immunofluorescence with BrdU-labeling and Hoechst staining in the dorsal hippocampus at the coronal plane including the caudal end of the splenium of the corpus callosum in valproic acid (VPA)-treated (left) and control (right) ferrets. **(B)** High magnification images of double immunofluorescence for calbindin D-28k and Sox2 with BrdU-labeling and Hoechst staining in the dentate gyrus of the dorsal hippocampus in VPA-treated (left) and control (right) ferrets. White open arrowheads indicate BrdU-labeled Sox2-immunopositive progenitors located in the molecular layer (ML), subgranular layer (SGL), and hilus. **(C)** High-magnification images of double immunofluorescence for NeuN and PH3 with BrdU-labeling and Hoechst staining in the dentate gyrus of the dorsal hippocampus in VPA-treated (left) and control (right) ferrets. White open arrowheads indicate BrdU-labeled PH3-immunopositive cells. White closed arrowheads indicate PH3-single stained cells. Yellow closed arrowheads indicate NeuN-single stained neurons in the hilus. **(D)** High-magnification images of double immunofluorescence for S100 and glial fibrillary acidic protein (GFAP) with BrdU-labeling and Hoechst staining in the dentate gyrus of the dorsal hippocampus in VPA-treated (left) and control (right) ferrets. White open arrowheads indicate BrdU-labeled S100-immunopositive cells. White closed arrowheads indicate S100-single stained neurons in the SGL and hilus Yellow closed arrowheads indicate GFAP-single stained astrocytes in the hilus.

The dorsal hippocampus in the coronal plane including the caudal end of the splenium of the corpus callosum was depicted by calbindin D-28k immunofluorescence with BrdU labeling and Hoechst staining in both VPA-treated and control ferrets (Figure 2A). In the dentate gyrus (DG), the subgranular layer was distinguishable by an array of calbindin D-28k-negative Hoechst-stained cells beneath a layer consisting of calbindin D-28k-positive granular neurons. To assess the effect of neonatal VPA exposure, the densities of cells defined by marker antigens for neuronal progenitors, neurons, and glial cells were estimated. There were no differences in the densities of calbindin D-28k-positive granular neurons in the DG granular layer and calbindin D-28k-negative Hoechst-stained cells in the DG subgranular layer between VPA-treated and control ferrets (Supplementary Figures 1A,B).

**FIGURE 3:**
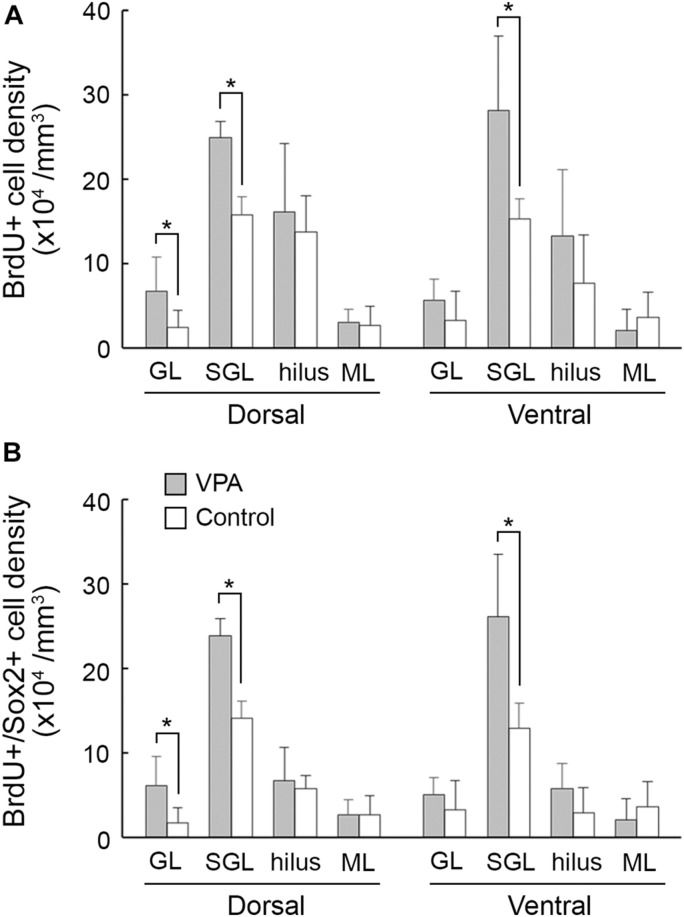
The 5-bromo-2-deoxyuridine (BrdU)-labeled cell density and BrdU-labeled Sox2-immunopositive progenitor density in the dentate gyrus of dorsal and ventral hippocampi in postnatal day (PD) 20 ferrets. **(A)** BrdU-labeled cell density. **(B)** BrdU-labeled Sox2-immunopositive progenitor density. Data are shown as mean ± SEM. Significance is indicated using Scheffe’s test at *P < 0.001; number of hippocampi = 8; GL, granular layer; ML, molecular layer; SGL, subgranular layer.

5-Bromo-2-deoxyuridine -labeled cells were found throughout the hippocampus of PD 20 ferrets and were particularly abundant in the DG (Figure 2A). A significant effect on treatments [*F*_(1,14)_ = 24.756, *P* < 0.001], but not hippocampal regions (dorsal/ventral), was observed in the density of BrdU-labeled cells in the DG subgranular layer by repeated-measures two-way ANOVA. *Post hoc* testing indicated significantly denser BrdU-labeled cells in VPA-treated ferrets compared to controls in both the dorsal and ventral hippocampi (Figure 3A). Significantly denser BrdU-labeled cells in VPA-treated ferrets compared to controls were also detected in the DG granular layer of the dorsal hippocampus (*P* < 0.001; Figure 3A), following a significant effect on treatment [*F*_(1,14)_ = 8.612, *P* < 0.05] by repeated-measures two-way ANOVA. This meant a slight disarrangement of the DG granular layer of the dorsal hippocampus by mismigration of the tracked neonatal DG progenitors, although this disarrangement was difficult to define optically. In the CA1 and CA3 fields, there was no difference in the BrdU-labeled cell density between VPA-treated and controls (Supplementary Figures 2A,C).

Sox2-positive progenitors were distributed widely throughout the hippocampus in both VPA-treated ferrets and controls on PD 20. Multiple immunofluorescence staining revealed the presence of BrdU labeling in Sox2-positive progenitors (Figure 2B). More than 70% of BrdU labeling appeared in Sox2-positive progenitors in the DG (Table 2) and CA1 and CA3 fields (Supplementary Tables 1, 2) in both groups. The density of BrdU-labeled Sox2-progenitors was estimated in the subregions of the dorsal and ventral hippocampi. Repeated-measures two-way ANOVA revealed a significant effect of treatment [*F*_(1,14)_ = 99.127, *P* < 0.001] and hippocampal regions (dorsal/ventral) [*F*_(1,14)_ = 13.057, *P* < 0.005], and these two interactions [*F*_(1,14)_ = 6.018, *P* < 0.05] in the DG subgranular layers. *Post hoc* testing indicated significantly denser BrdU-labeled Sox2-progenitors in VPA-treated ferrets than in controls in the DG subgranular layer of both the dorsal and ventral hippocampi (Figure 3B). In the DG granular layer, significantly denser BrdU-labeled Sox-2-positive progenitors in VPA-treated ferrets compared to controls were detected in the dorsal hippocampus (*P* < 0.001; Figure 3B), following a significant effect on treatment [*F*_(1,14)_ = 7.118, *P* < 0.05] by repeated-measures two-way ANOVA. In other hippocampal regions examined, including the CA1 and CA3 fields, there was no difference in the BrdU-labeled Sox-2-positive progenitor density between VPA-treated and control ferrets (Supplementary Figures 2B,D).

**TABLE 2:** Percentages of cells immunostained for various markers in BrdU-labeled cells in dentate gyrus of hippocampus in VPA-treated ferrets on PD 20.

	Dorsal hippocampus		Ventral hippocampus	
	VPA	Control	VPA	Control
**Granular layer**				
% of Sox2+	90.0%(18/20)	71.4%(5/7)	88.2%(15/17)	100.0%(10/10)
% of C28k+	0%(0/20)	0%(0/7)	0%(0/17)	0%(0/10)
% of S100+	0%(0/25)	0%(0/23)	10.0%(2/10)	0%(0/13)
% of GFAP+	0%(0/25)	0%(0/23)	0%(0/10)	0%(0/13)
**Subgranular layer**				
% of Sox2+	96.0%(72/75)	89.4%(42/47)	92.9%(78/84)	84.8%(39/46)
% of S100+	15.3%(17/111)	7.0%(5/71)	10.9%(10/92)	2.1%(1/47)
% of GFAP+	0%(0/111)	0%(0/71)	0%(0/92)	0%(0/46)
**Hilus**				
% of Sox2+	89.1%(41/46)	85.4%(35/41)	89.7%(35/39)	78.3%(18/23)
% of NeuN+	0%(0/53)	5.6%(1/18)	0%(0/31)	6.3%(1/16)
% of S100+	6.5%(2/31)	4.2%(1/24)	4.8%(1/21)	0%(0/8)
% of GFAP+	0%(0/31)	0%(0/24)	0%(0/21)	0%(0/8)
**Molecular layer**				
% of Sox2+	88.9%(8/9)	100.0%(8/8)	100.0%(6/6)	100.0%(11/11)
% of NeuN+	0%(0/24)	0%(0/16)	0%(0/20)	0%(0/10)
% of S100+	8.3%(1/12)	13.3%(2/15)	0%(0/10)	0%(0/5)
% of GFAP+	0%(0/12)	0%(0/15)	0%(0/10)	0%(0/5)

*Percentages are calculated by summing each immunolabeled cell counted within all ROIs from eight cerebral hemispheres. The number of each labeled cell for calculating the percentages is shown in parentheses. There were no significant difference in the incidence in any subregions of the dentate gyrus of both dorsal and ventral hippocampus between VPA-treated and control ferrets.*

Multiple immunofluorescence staining was conducted using neuronal and glial markers, that is, granular neurons in the DG and pyramidal neurons in the CA fields (calbindin D-28k-positive), neurons (NeuN-positive), and glial cells (S100-positive or GFAP-positive). NeuN-positive neurons were distributed sparsely in the hilus and molecular layer of the DG (Figure 2C), the strata radiatum and oriens of the CA1 and the CA3, and also aligned into the DG granular layer and the CA pyramidal layer. Calbindin D-28k immunostaining also appeared predominantly in DG granular neurons and CA pyramidal neurons (Figures 2A,B). While 5.9% of BrdU-labeled cells were unexpectedly NeuN-positive in the CA3 stratum radiatum, almost all NeuN-positive neurons and calbindin D-28k granular/pyramidal neurons in the hippocampus were not labeled with BrdU in both VPA-treated and control ferrets (Figures 2B,C, Table 2 and Supplementary Tables 1, 2).

While S100-positive cells were distributed throughout the hippocampus, their alignment at the bottom of the DG subgranular layer was striking (Figure 2D). S100 immunostaining was found in BrdU-labeled cells in the range of 0–15.3% in VPA-treated ferrets and from 0 to 13.3% in controls (Table 2). There was no difference in the incidence of S100 immunostaining in BrdU-labeled cells in any DG subregion between VPA-treated and controls. In contrast, no BrdU-positive cells expressed S100 immunostaining in the CA1 and CA3 fields in either group (Supplementary Tables 1, 2).

GFAP immunostaining mainly appeared in astrocytes sparsely distributed throughout the hippocampus (yellow closed arrowhead in Figure 2D) and was not observed in radial glia-like cells aligned in the DG granular through subgranular layers (Figure 2D), as seen in the adult ferret hippocampus (Supplementary Figures 3A,B). There were no BrdU-labeled GFAP-positive astrocytes in the hippocampal subregions, including the DG, except the stratum oriens in the CA1 and CA3 fields. GFAP-positive astrocytes were found in BrdU-labeled cells of the CA1 stratum oriens at 5.9% in the ventral hippocampus of VPA-treated ferrets and 12.5% in the dorsal hippocampus of control ferrets (Supplementary Table 1). In the CA3 stratum oriens, 21% of BrdU-labeled cells were GFAP-positive astrocytes in the dorsal hippocampus of VPA-treated ferrets (Supplementary Table 2). In any case, there was no difference in the incidence of GFAP-positive astrocytes in BrdU-labeled cells in all hippocampal subregions examined between VPA-treated and control ferrets.

Notably, immunostaining for PH3, a specific marker for mitosis, was found in a very small population of BrdU-labeled cells in the hippocampus (Figure 2C). Only 0.2 and 0.5% of BrdU-labeled cells distributed throughout the hippocampus were PH3-immunopositive in VPA-treated and control ferrets, respectively. The co-presence of PH3 immunostaining with BrdU labeling indicates the possibility that Sox2-positive progenitors distributed throughout the hippocampus may have mitotic potency.

### Immunohistochemical Analysis of Postnatal Day 7 Ferret Premature Hippocampi

**FIGURE 4:**
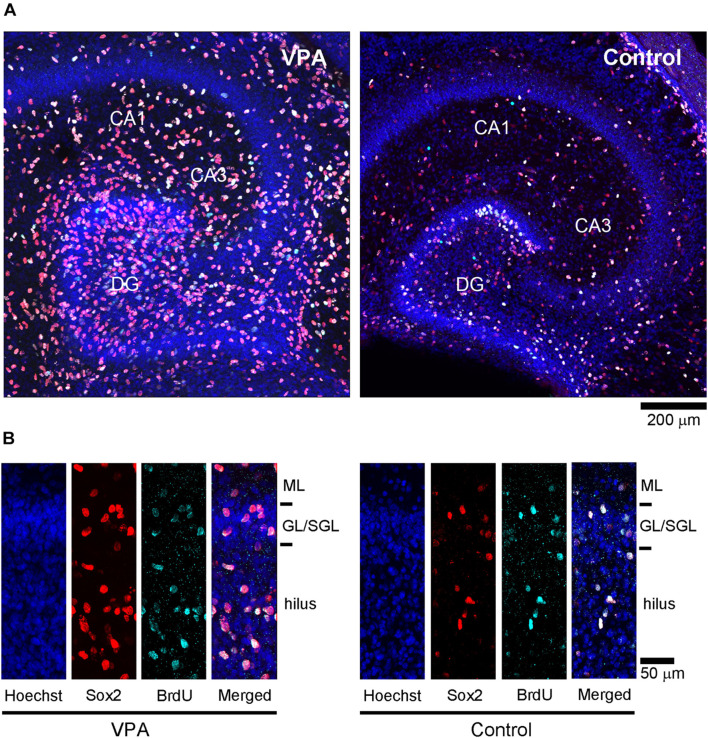
Sox2 immunofluorescence with 5-bromo-2-deoxyuridine (BrdU)-labeling and Hoechst staining in premature hippocampus of postnatal day (PD) 7 ferrets. **(A)** Low-magnification images in the premature hippocampi of valproic acid (VPA)-treated (left) and control (right) ferrets. **(B)** High-magnification images of Sox2 immunofluorescence with BrdU-labeling and Hoechst staining in the dentate gyrus in VPA-treated (left) and control (right) ferrets. GL, granular layer; ML, molecular layer; SGL, subgranular layer.

**FIGURE 5:**
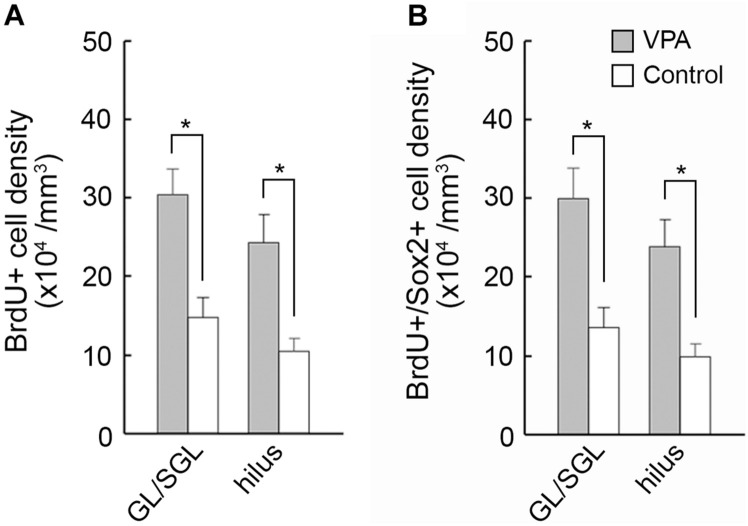
The 5-bromo-2-deoxyuridine (BrdU)-labeled cell density and BrdU-labeled Sox2-immunopositive progenitor density in the dentate gyrus of premature hippocampi in postnatal day (PD) ferrets. **(A)** BrdU-labeled cell density. **(B)** BrdU-labeled Sox2-immunopositive progenitor density. Data are shown as mean ± SEM. Significance is indicated using Scheffe’s test at *P < 0.001; number of hippocampi = 8; GL, granular layer; SGL, subgranular layer.

Immunofluorescence staining for Sox2 with BrdU labeling was performed in the premature hippocampus of VPA-treated ferrets and controls on PD 7, 2 h following BrdU injection simultaneously with the last VPA injection. BrdU-labeled Sox2-positive progenitors were abundant, particularly in the granular and subgranular layers and hilus of the DG in the premature hippocampus (Figure 4). As it was difficult to distinguish the border between granular and subgranular layers in the premature DG, the density of BrdU-labeled Sox2-positive progenitors was estimated in areas including either the granular layer or subgranular layer or the hilus. BrdU-labeled cells were significantly denser in both the granular/subgranular layers and hilus in VPA-treated ferrets than in controls (Figure 5). Sox2-positive progenitors were found in BrdU-labeled cells at 97.5% and 88.2% in the granular/subgranular layers and 94.6% and 91.7% in the hilus of VPA-treated and control ferrets, respectively (Table 3). The density of BrdU-labeled Sox2-positive progenitors was significantly greater in both the granular/subgranular layers and hilus of VPA-treated ferrets than those of controls (Figure 5). Thus, VPA exposure facilitated the proliferation of Sox2-positive progenitor pools in the DG of the premature hippocampus.

**TABLE 3:** Percentages of Sox2-immunopositive progenitors in BrdU-labeled cells in dentate gyrus of hippocampus in VPA-treated ferrets on PD 7.

	VPA	Control
Granular/subgranular layers	95.7% (67/70)	88.2% (30/34)
Hilus	94.6% (53/56)	91.7% (22/24)

*Percentages are calculated by summing each immunolabeled cell counted within all ROIs from six cerebral hemispheres. The number of each labeled cell for calculating the percentages is shown in parentheses. There were no significant difference in the incidence in any subregions of the dentate gyrus of hippocampus between VPA-treated and control ferrets.*

## Discussion

The effect of VPA on the developing hippocampus has been studied mainly during neural tube development. These findings are inconsistent; although a previous study has reported an increased density of neurons in the DG and CA fields in rats (Edalatmanesh et al., 2013), other studies have reported enhanced neurogenesis with decreased neuronal progenitor pools in mice (Juliandi et al., 2015) and reduced expression of GAD 67, an isoform of GABA synthetic enzyme, in rats (Hou et al., 2018; Win-Shwe et al., 2018). In contrast, there have been a few studies regarding the effect of VPA on the hippocampus during neonatal periods. Neonatal VPA exposure induced cell proliferation rather than neuronal differentiation in the hippocampal DG 24 h after VPA injection in rat infants (Wang et al., 2016). The current investigation consistently showed proliferation of Sox2-positive progenitors in the hippocampal DG of PD 7 ferrets immediately following VPA injection. The putative precursors of granular neurons appeared massively in the DG during the first week of postnatal age in mice (Nicola et al., 2015; Hevner, 2016). Therefore, stable results may be obtained by neonatal VPA exposure targeting DG progenitors rather than prenatal VPA exposure targeting neural tube development. It has been reported that DG progenitors are changed from embryonic-type to adult-type during early postnatal ages in mice (Matsue et al., 2018). VPA has a proliferative effect on neonatal DG progenitors (embryonic-type) (Wang et al., 2016; present results); however, it facilitates adult neurogenesis of the DG progenitors (adult-type) (Hsieh et al., 2004). It may be interesting to note the factors responsible for the diverse effects of VPA on embryonic and adult forms of DG progenitor cells. Regarding the adult type, VPA-facilitated adult neurogenesis is mediated by inhibition of histone deacetylase activity in rats (Hsieh et al., 2004). One of the factors related to facilitating the proliferation of the embryonic-type DG progenitors may be the Wnt/β-catenin pathway, which was reportedly upregulated in the hippocampus of VPA-treated rat fetuses (Wang et al., 2010).

The present study tracked the DG progenitors of PD 7 ferret infants by labeling them with BrdU and examined their fates on PD 20 using neuronal and glial markers. Although tracked cells could not be fully characterized, they expressed Sox2 in the majority, but not neural (NeuN and calbindin D-28k) and glial (GFAP and S100) markers examined in both VPA-treated and control ferrets. Notably, the density of tracked cells expressing Sox2 in the subgranular layer was significantly increased by neonatal VPA exposure. This suggests that VPA promotes the proliferation of embryonic-type DG progenitors, resulting in increased adult-type DG progenitors, mainly in the DG subgranular layer. In contrast, GFAP-positive radial processes vertically across the DG granular and subgranular layers were found in young adult ferrets (PD 90), but not in prepubertal immature ferrets (PD 20). The DG progenitors had GFAP-positive radial processes, even if they were embryonic-type, in humans (Stagaard Janas et al., 1991), rats (Rickmann et al., 1987; Li et al., 2011), and mice (Matsue et al., 2018). Therefore, the chemical characteristics of embryonic and adult DG progenitors may be diverse among mammalian species. Embryonic DG progenitors may lack GFAP expression specifically in carnivores, and are still low in PD 20 ferrets.

The cerebral cortex was thickened and expanded its width at the posterior one-third when ferrets were exposed to VPA by an identical administration schedule to the current investigation (Sawada et al., 2021). The substantial effect of neonatal VPA exposure on the ferret hippocampus may increase adult-type DG progenitors in the DG subgranular layer in the present study. Prenatal VPA exposure has a long-term effect on DG progenitors, impairing adult neurogenesis and hippocampus-dependent learning and memory in mice (Juliandi et al., 2015). Impairment of social behaviors has been reported in ferrets exposed to neonatal VPA (Krahe et al., 2016), but not in hippocampus-related behavioral changes. The current findings revealed mitotic potency on tracked neonatal DG progenitors in both VPA-treated ferrets and controls on PD 20. Further studies are needed to evaluate neurogenesis of the tracked neonatal DG progenitors in the subgranular layer and hippocampal-related behaviors in VPA-treated ferrets.

## Conclusion

Prenatal and neonatal exposure to VPA induces ASD-like behavioral abnormalities in various mammalian species (Miyazaki et al., 2005; Yochum et al., 2008; Mychasiuk et al., 2012; Favre et al., 2013; Burenkova et al., 2014; Yasue et al., 2018), including ferrets (Krahe et al., 2016). These behavioral abnormalities are believed to be associated with cerebral cortical abnormalities that have been reported as the effect of developmental VPA exposure in ferrets (Sawada et al., 2021) and other mammalian species (Wood et al., 2014; Fujimura et al., 2016; Lauber et al., 2016). In contrast, prenatal and neonatal VPA exposure can alter the development of various brain regions other than the cerebral cortex. The current findings revealed that neonatal VPA exposure alters the progenitors in the DG subgranular layers, which may be involved in hippocampal adult neurogenesis and its related behaviors. This will be helpful for a comprehensive understanding of brain abnormalities induced by neonatal VPA exposure.

## Data Availability Statement

The original contributions presented in the study are included in the article/Supplementary Material, further inquiries can be directed to the corresponding author.

## Ethics Statement

The animal study was reviewed and approved by Institutional Animal Care and Use Committees of Tsukuba International University.

## Author Contributions

All authors had full access to all the data in the study and take responsibility for the integrity of the data and the accuracy of the data analysis. KS: study concept and design. KS, SK, and IA: acquisition of data and critical revision of the manuscript for important intellectual content. KS and IA: analysis and interpretation of data, drafting of the manuscript, and obtained funding.

## Conflict of Interest

The authors declare that the research was conducted in the absence of any commercial or financial relationships that could be construed as a potential conflict of interest.

## Publisher’s Note

All claims expressed in this article are solely those of the authors and do not necessarily represent those of their affiliated organizations, or those of the publisher, the editors and the reviewers. Any product that may be evaluated in this article, or claim that may be made by its manufacturer, is not guaranteed or endorsed by the publisher.
