# Supplementary material for: The Proliferation of Dentate Gyrus Progenitors in the Ferret Hippocampus by Neonatal Exposure to Valproic Acid
Source: Front Neurosci. 2021 Sep 28;15:736313. doi: 10.3389/fnins.2021.736313 (PMC8505998; doi:10.3389/fnins.2021.736313)
Supplement: Supplementary file 1 [file Data_Sheet_1.pdf]

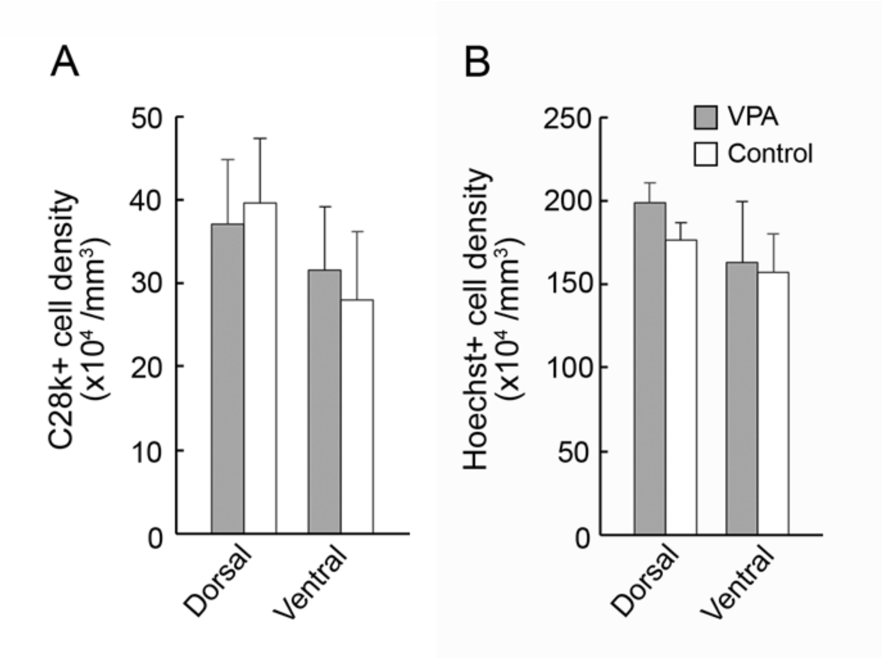

Supplemental Figure 1. Densities of calbindin D-28k-positive granular neurons and calbindin D-28k-negative Hoechst-stained cells in the dentate gyrus of the dorsal and ventral hippocampi in ferrets on postnatal day 20. (A) Calbindin D-28k-positive granular neuron density in the granular layer. (B) Calbindin D-28k-negative Hoechst-stained cells in the subgranular layer.
