# Supplementary material for: The Proliferation of Dentate Gyrus Progenitors in the Ferret Hippocampus by Neonatal Exposure to Valproic Acid
Source: Front Neurosci. 2021 Sep 28;15:736313. doi: 10.3389/fnins.2021.736313 (PMC8505998; doi:10.3389/fnins.2021.736313)
Supplement: Supplementary file 2 [file Data_Sheet_2.pdf]

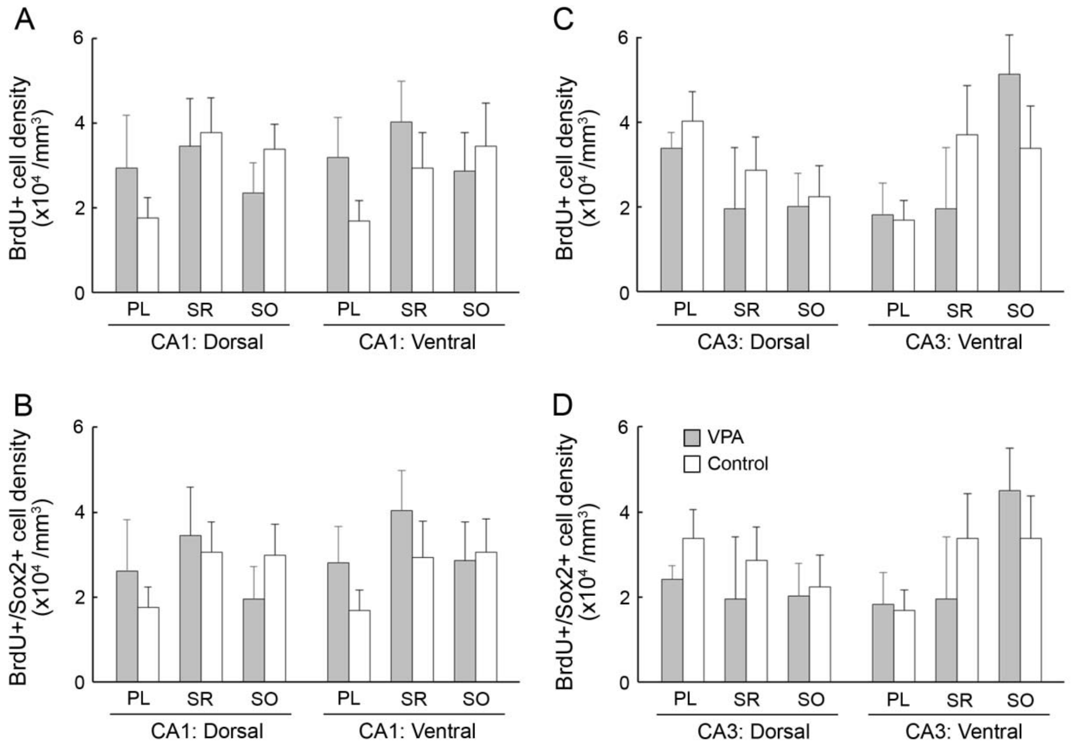

Supplementary Figure 2. The 5-Bromo-2-deoxyuridine (BrdU)-labeled cell density and BrdU-labeled Sox2-immunopositive progenitor density in CA1 and CA3 fields of dorsal and ventral hippocampi in ferrets on postnatal day 20. (A) BrdU-labeled cell density in the CA1 field. (B) BrdU-labeled Sox2-immunopositive progenitor density in the CA1 field. (C) BrdU-labeled cell density in the CA3 field. (D) BrdU-labeled Sox2-immunopositive progenitor density in the CA3 field. Data are shown as mean ± standard error of the mean (SEM). The number of hippocampi = 8; PL, pyramidal layer; SR, stratum radiatum; SO, stratum oriens.
