# Supplementary material for: The Proliferation of Dentate Gyrus Progenitors in the Ferret Hippocampus by Neonatal Exposure to Valproic Acid
Source: Front Neurosci. 2021 Sep 28;15:736313. doi: 10.3389/fnins.2021.736313 (PMC8505998; doi:10.3389/fnins.2021.736313)
Supplement: Supplementary file 3 [file Data_Sheet_3.pdf]

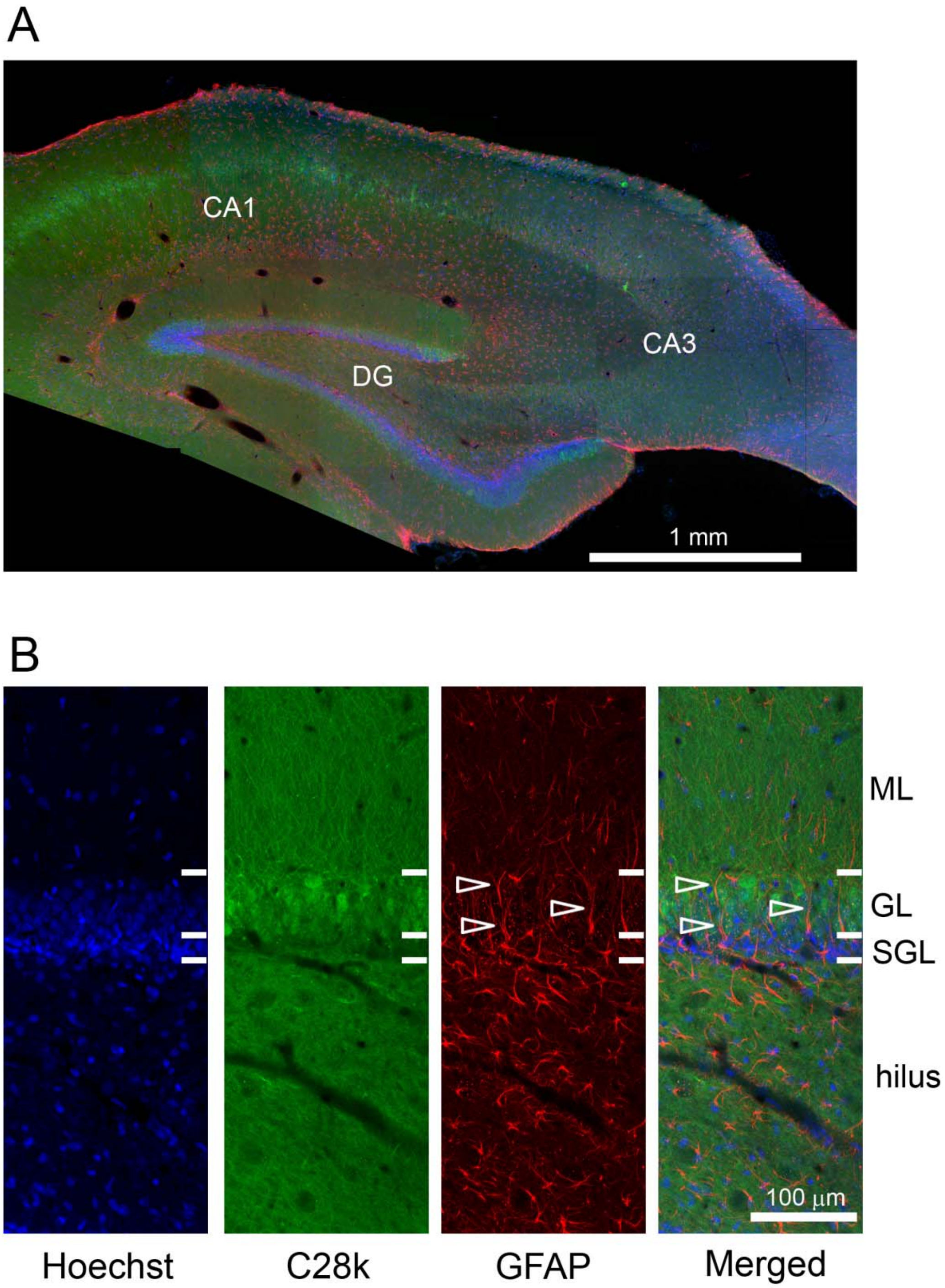

Supplementary Figure 3. Double immunofluorescence for Calbindin D-28k and glial fibrillary acidic protein (GFAP) with Hoechst staining in the dorsal hippocampus of the young adult ferret (postnatal day 90). (A) Low magnification image. (B) High magnification image. Open arrowheads indicate GFAP-immunopositive radial glia-like cells aligned in the granular (GL) through subgranular (SGL) layers. These cells are not observed in ferret hippocampi on postnatal day 20. ML, molecular layer.
