# Supplementary material for: The Proliferation of Dentate Gyrus Progenitors in the Ferret Hippocampus by Neonatal Exposure to Valproic Acid
Source: Front Neurosci. 2021 Sep 28;15:736313. doi: 10.3389/fnins.2021.736313 (PMC8505998; doi:10.3389/fnins.2021.736313)
Supplement: Supplementary file 5 [file Table_2.pdf]

Supplementary Table 2. Percentages of cells immunostained for various markers in BrdU-labeled cells in hippocampal CA3 field in VPA-treated ferrets on PD 20.

	Dorsal hippocampus				Ventral hippocampus			
	VPA		Control		VPA		Control	
Pyramidal layer								
% of Sox2+	100.0%	(4/4)	83.3%	(10/12)	100.0%	(5/5)	100.0%	(5/5)
% of C28k+	0%	(0/4)	0%	(0/12)	0%	(0/5)	0%	(0/5)
% of S100 +	0%	(0/11)	0%	(0/7)	0%	(0/10)	0%	(0/6)
% of GFAP+	0%	(0/11)	0%	(0/7)	0%	(0/10)	0%	(0/6)
Stratum radiatum								
% of Sox2+	100.0%	(5/5)	100.0%	(8/8)	100.0%	(5/5)	90.0%	(9/10)
% of NeuN+	5.9%	(1/17)	0%	(0/9)	0%	(0/13)	0%	(0/8)
% of S100 +	0%	(0/11)	0%	(0/10)	0%	(0/8)	0%	(0/7)
% of GFAP+	0%	(0/11)	0%	(0/10)	0%	(0/8)	0%	(0/7)
Stratum oriens								
% of Sox2+	100.0%	(6/6)	100.0%	(6/6)	86.7%	(13/15)	100.0%	(10/10)
% of NeuN+	0%	(0/28)	0%	(0/13)	0%	(0/14)	0%	(0/13)
% of S100 +	0%	(0/14)	0%	(0/11)	0%	(0/10)	0%	(0/8)
% of GFAP+	21.4%	(3/14)	0%	(0/11)	0%	(0/10)	0%	(0/8)

Percentages were calculated by summing each immunolabeled cell counted within all ROIs from 8 cerebral hemispheres. The number of each labeled cell for calculating the percentages is shown in parentheses. There were no significant difference in the incidence in any subregions of the CA3 field of both dorsal and ventral hippocampus between VPA-treated and control ferrets
